# Acute pancreatitis revealing an isolated hydatid pancreatic cyst simulating a pseudocyst: A case report

**DOI:** 10.1016/j.radcr.2023.01.064

**Published:** 2023-03-07

**Authors:** Ihssane Afilal, Widad Abbou, Khaoula Oukrid, Narjisse Aichouni, Siham Nasri, Imane Kamaoui, Imane Skiker

**Affiliations:** a Department of Radiology, Mohammed VI University Hospital, Bouskoura, Morocco; b Faculty of Medicine, University Mohammed First, BP 4806 Oujda University 60049, Oujda, Morocco

**Keywords:** Pancreatitis, Pseudocyst, Hydatid cyst, Echinococcus, MRI, Diagnosis

## Abstract

Hydatid disease is a worldwide public health problem, especially in endemic countries, caused by the larval stage of Echinococcus granulosis. The pancreatic location of this disease is exceptional, representing only 1% of all possible locations, making this a widely misdiagnosed entity. We report a case of a 42-year-old man with a history of alcoholism and recurring abdominal pain, who presented to the emergency department with acute pancreatitis revealing a hydatid pancreatic cyst mimicking as a pseudocyst of the pancreas. The diagnosis was established using computed tomography and magnetic resonance imaging.

## Introduction

Pancreatic hydatid cysts are an exceptional form of hydatid disease, and isolated involvement has rarely been reported [1]. This explains the diagnostic difficulty of this entity. The most common locations of hydatid cysts are liver (50%-77%), lung (15%-47%), spleen (0.5%-8%), and kidney (2%-4%) [2].

We describe a case of a 42-year-old man admitted to the emergency department with typical pancreatitis symptoms, for which the initial CT scan reasonably evoked the differential diagnosis of a pseudocyst given the history of alcoholism and recurring abdominal pain, and the absence of typical radiological hydatid cyst characteristics. However, a subsequent MR imaging confirmed the diagnosis of a hydatid pancreatic cyst. This case report highlights the importance of considering this diagnosis in case of cystic lesions of the pancreas, especially in endemic areas.

## Patient and observation

**Fig. 1(A):**
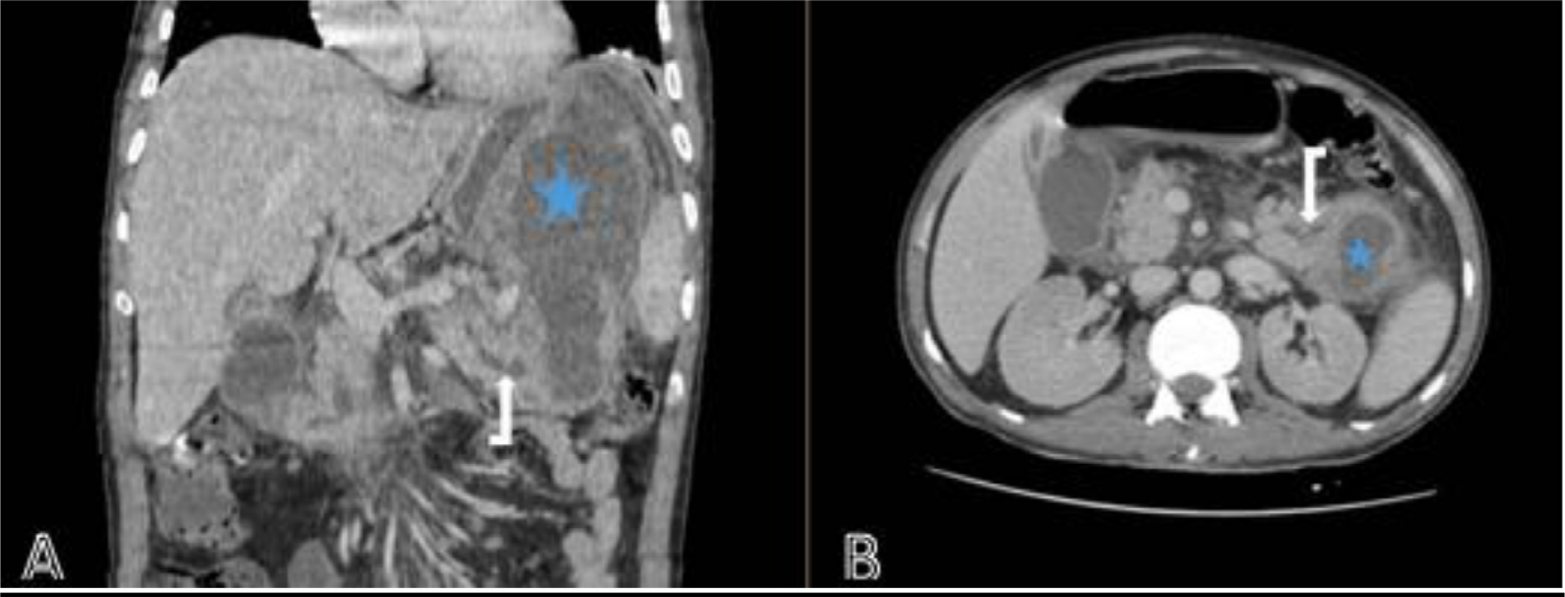
Coronal section (B) axial section of a contrast enhanced abdominal CT scan revealing signs of stage E pancreatitis associated to a cystic mass (blue star) extending between the pancreatic tail and the gastric region measuring approximately 15 × 6 cm. This mass seems to communicate with the Wirsung pancreatic duct which is dilated (white arrow).

**Fig. 2(A):**
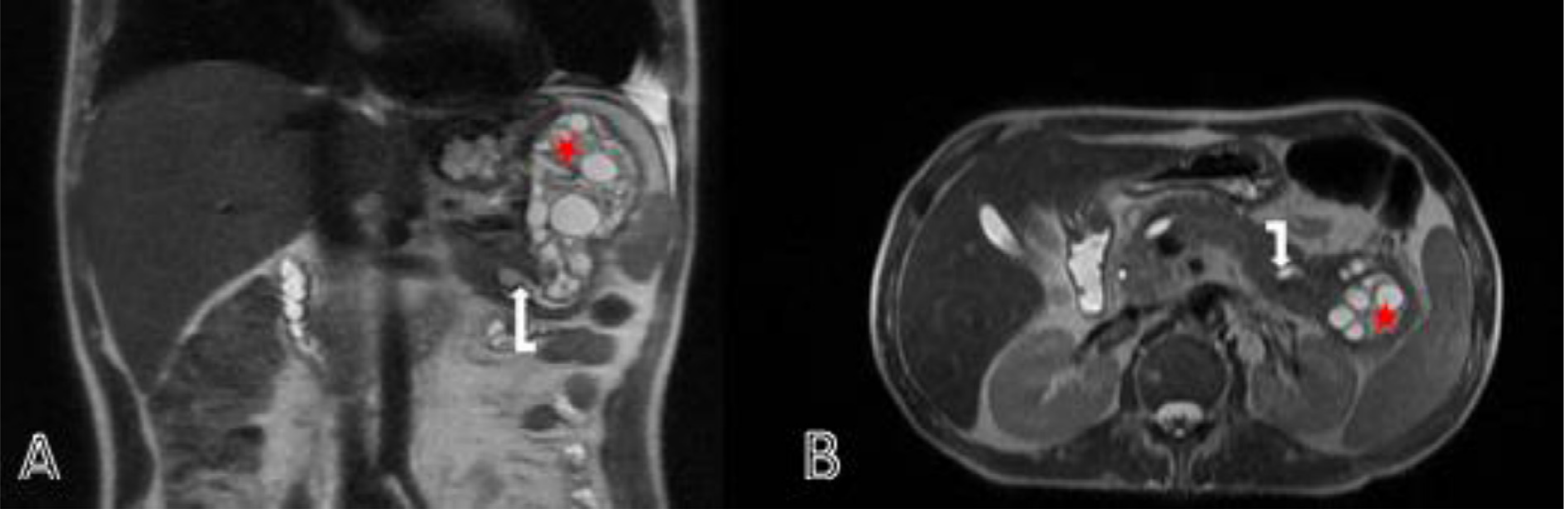
Coronal section (B) axial section of a Bili-MRI showing multiple daughter cysts inside the pancreatic cystic mass (red star), consistent with a type III hydatid cyst of the GHARBI classification. This cystic mass is fistulised in the Wirsung pancreatic duct (white arrow) causing a stage E pancreatitis.

**Fig. 3(A-C):**
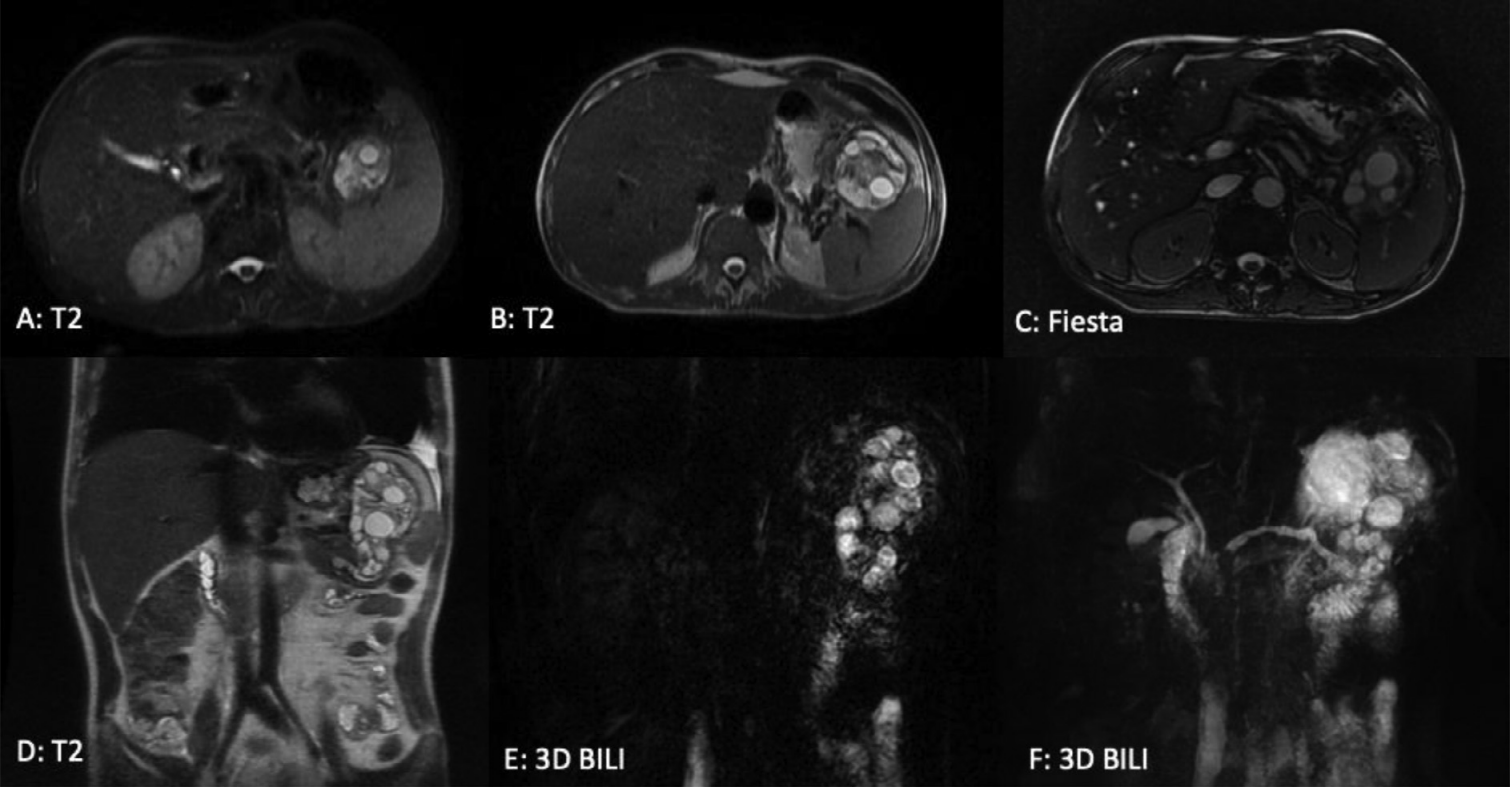
axial section (D-F) coronal section of a Bili-MRI showing a hydatid cyst causing a stage E pancreatitis.

A 42-year-old male, with a history of alcoholism and recurring abdominal pain, originating from an endemic area of hydatid disease in North Africa, was admitted to the emergency department, for an acute onset of severe central epigastric pain radiating through the back and exacerbated in supine position, associated to nausea for 3 days. Moreover, he did not have any significant prior medical history. Clinical examination revealed a palpable mass occupying the left hypochondrium and the epigastrum with firm consistency. A standard lab report was ordered revealing elevated lipasemia of more than 20 times the normal range, a moderate cytolysis, high white blood count and CRP levels, with a mildly altered renal function (Table 1). Hydatid serology was not ordered at this juncture. Seeing as the patient was at 72 hours of the first onset of pancreatitis type pain, a contrast enhanced abdominal CT scan was initially ordered, revealing a well-defined cystic mass centered on the tail of the pancreas measuring approximately 155 × 65 mm, communicating with the Wirsung pancreatic duct that was dilated as well as a fistula in the adjacent gastric wall. The scan also showed an enlarged homogeneously enhanced pancreatic gland with extensive peripancreatic fat stranding and fluid collections, consistent with stage E pancreatitis (Fig. 1). Considering the history of alcoholism and recurring episodes of epigastric pain, as well as the lack of pathognomonic radiological features of hydatid infection, the diagnosis of a pseudocyst associated to an acute pancreatitis was given as the main differential diagnosis. The other differential considered was intraductal papillary mucinous tumor seeing as the cyst presented a communication with the main pancreatic duct. Moreover, an MRI was ordered to further analyze this cystic mass and its communication with the pancreatic ducts, this showed multiple daughter cysts with variable shapes and sizes occupying almost the entire volume the mother cyst that were otherwise indistinguishable on CT scan (Figs. 2, 3). This cystic mass is fistulized in the Wirsung pancreatic duct, causing the pancreatitis. The patient underwent surgery for primary pancreatic hydatid disease, a distal pancreatectomy with splenectomy was performed and the main duct was cleaned from scolices. Histopathological examination further confirmed the diagnosis of a hydatid cyst. Albendazole therapy 800mg/day was prescribed for 3 months after the surgery, and the weeks following the operation were without complications.

**Table 1:** Laboratory findings.

Labs	Value	Reference range
Lipasemia	1500	0-70 U/L
CRP	320	<5 mg/L
WBC count	65.29	4.0-10.0 thousand/mm^3^
Hemoglobin	10.7	12-16 g/dL
Alanine aminotransferase (ALT)	241	0-42 U/L
Aspartate aminotransferase (AST)	403	0-37 U/L
Total bilirubine	14	12 mg/dl
Creatinine	18.28	6-13 mg/L

## Discussion

Hydatidosis is secondary to the development of the larval form of Echinococcus granulosus in humans. Hydatid cysts can touch any organ or structure of the body. However, the pancreatic location has a prevalence of less than 1% [2]. It is isolated in 90% of cases with a predilection for the cephalic part of the pancreas (57%) [1]. Humans are accidental hosts, as infection occurs by ingesting food contaminated with Echinococcus eggs. Hematogenous dissemination is hypothesized to be the most common mode of spread to pancreas, moreover lymphatic spread from intestinal mucosa, passage through the biliary system, and direct passage from the pancreatic veins and retroperitoneal dissemination are other probable contamination modalities [3]. The clinical manifestations of pancreatic hydatid cyst are nonspecific and depend on the size, the site and complications of the cyst such as rupture, abscess, biliary or digestive fistula, pancreatitis or compression of adjacent structures [4]. Symptoms can include chronic epigastric pain, cholestatic jaundice (cephalic location), a palpable abdominal mass, portal vein thrombosis, or even anaphylactic choc. In our case, the revealing symptoms were typical pancreatitis pain and a palpable mass. Computed tomography and magnetic resonance are able to reveal the cystic nature of the mass but the characteristic radiological findings described for hydatid cysts are rarely present [5]. However certain signs could guide the diagnosis such as the “water lily sign” in case of a detachment of internal serpentine undulant membranes, the “beehive sign” in case of the presence of daughter cysts inside a larger mother cyst and a thick calcified wall of the cyst, and the association of other more evident locations of hydatid infection such as liver and lung. Endoscopic ultrasound guided aspiration of pancreatic cystic fluid combined with cytological and biochemical analysis can help exclude cystic neoplasms and pseudocysts of the pancreas [5]. The differential diagnosis includes pseudocysts that are usually associated to a history of pancreatitis or trauma. Cystadenoma and cystadenocarcinoma, which are enhanced after contrast and usually have thick septas. Intraductal papillary mucinous tumors which are usually associated to dilation of the main pancreatic duct over 5 mm and rarely present calcifications. The management of cystic echinococcosis is mainly surgical and depends on the location of the cyst and the presence of fistulas or other complications, other treatment options include antiparasitic drug therapy and percutaneous treatment [6]. This technique consists of Puncture-Aspiration-Injection of scolicidal agent and Reaspiration (PAIR), can be used for diagnosis and treatment and has been proven to be an effective replacement to surgery with lower rates of recurrence and mortality [7]. Open surgery is the classical approach to treat hydatid cysts of the pancreas, especially in case of complicated cysts and when percutaneous treatment is not available [3], [4], [5], [6]. Evacuation of the cyst and defacement of the residual cavity are the aims of the surgical therapy [8]. Medical therapy consisting of albendazole 10mg/kg/day 4 weeks before and after the procedure reduces the risk of recurrence [2].

Isolated hydatid cyst of the pancreas is a very rare entity. Our intention in presenting this case report is to highlight the possibility of a pancreatic location of a hydatid infection masquerading as a pseudocyst or a cystic neoplasm. In patients from endemic areas a pancreatic hydatid cyst should always be considered as a differential diagnosis.

## Conclusion

Hydatid cyst of the pancreas is an extremely rare and widely misdiagnosed entity, especially in case of an isolated cystic lesion simulating a pseudocyst or cystic neoplasm. Hydatid cyst should be considered as one of the differential diagnosis in pancreatic cystic lesions, as well as other organs, especially in patients originating from endemic countries. The management of this entity includes surgery, percutaneous techniques (PAIR) and antiparasitic drug therapy.

## Patient consent

Written informed consent for publication of this case study was obtained from the patient.
